# Domestic

**Published:** 1841-01-30

**Authors:** 


### DOMESTIC.

*Geneva Medical College.*—One hundred and twenty-six students have attended the course at this institution during the current session.

*Hospitals of Paris.* By M. L. Linton, M. D.

[The following interesting sketch of the Parisian Hospitals, is contained in a letter from Paris, published in the January number of the Western Journal of Medicine and Surgery.]

"The Hotel Dieu is not only the most ancient hospital of Paris, but perhaps of Europe. It was founded in the year 669, by St. Laundry, Bishop of Paris. It was intended for every variety of disease, all ages, sexes, conditions, countries and religions; its device being '*Medicus et hospes.*' The number of sick in this establishment has always been very great. It rose in 1709 to 9,000. It is said, or rather written, that during the middle ages it was not uncommon to see fifteen patients in one bed,—the dying, the dead, and the convalescent! The wards badly lighted, were worse ventilated. Diseases, contagious and non-contagious, medical and surgical, women dying with puerperal fever, and those under the pains of parturition, the sane and the insane, were promiscuously crowded together, and the surgical operations a spectacle for the whole.

Under these circumstances it is not at all wonderful, that Hotel Dieu was so insalubrious; and that its mortality was nearly twenty-five per cent. This wretched state of things continued, with some trivial modifications, until the revolution of 1789. Since that epoch it has undergone successive and important improvements; other hospitals have been established, and a proper distribution made of the sick. The insane were sent to Salpétrière and Bicêtre—those affected with venereal diseases were assigned to the Venereal Hospital, and those with diseases of the skin to the Hospital of St. Louis. Special hospitals for infants and lying-in women were also established, and those of La Charité and La Pitié enlarged. By these vast additions to the means of meeting the demands of the times, the population of Hotel Dieu was reduced to about 750—the ordinary number at present. Comfortable beds on iron bedsteads furnished with curtains, now fill its airy, neat, and extensive wards; and a convenient distribution is made of its inmates. The mortality of Hotel Dieu at this time is about one in nine, and the mean expense incurred for each patient, a little less than thirty-four cents per diem,—far less than when its accommodations were far worse. Those who from time to time have given an account of Hotel Dieu, attribute its mortality in part to its proximity to the central office of the hospitals, to which all applicants for admission are obliged to go for examination and authority to enter. Hotel Dieu being nearest, the worst cases, say they, are sent to it. Its principal physicians are Majendie, Récamier, and Chomel. Of Majendie it is not necessary to say much. He is known to all the world as an able physiologist, and (cruel?) vivisector. I have frequently attended his lectures and physiologico-pathological demonstrations. He is a short, strongly built little man, aged about fifty years, and a tolerably good, though not a fluent lecturer. His method is simply to pronounce his profession of faith, and then bring forward, or rather drag forward, as proofs, a parcel of poor dogs, that howlingly and unwillingly confirm it with their blood. Does there exist an imperious necessity for such martyrs?

Récamier has great reputation as a physician. Chomel is one of the first pathologists of the age. The surgeons of Hotel Dieu are Roux, Breschet, and Blandin. Roux is the chief, being the successor of Dupuytren. He is a robust, white haired old gentleman of about sixty, and perhaps the most adroit operator in the capital, though by no means a good lecturer. He speaks too rapidly to be understood by foreigners, and hence is not so much followed by them as Velpeau, whese enunciation is slow and distinct. Breschet and Blandin are known as anatomists.

The Hôpital de la Charité contains about 500 beds, and may be considered as second in importance to Hotel Dieu. For the year 1837 its mortality, in the medical department, was one in nine; in the surgical, one in twenty-two—the mean cost for each patient, per diem, thirty cents—the number of sick admitted, 8,157, and the mean duration of their stay, seventeen days. Its physicians are Andral, Rayer, Bouillaud, Fouquier, and Bailly. Andral is a serious, thoughtful looking man, aged about fifty years, and unsurpassed as a lecturer. Rayer is rather youthful in appearance and very large, resembling somewhat his recent large work on diseases of the skin. Bouillaud's person is tall and slight; his forehead is high and expansive. He is an ardent disciple of Broussais, and distinguished for a voluminous and well written work on the diseases of the heart. Fouquier is as large, almost, as Rayer. He is an old man and a popular physician. Bailly is known as the introducer of several articles into the materia medica. Velpeau is the principal surgeon. His age is between forty and fifty, his hair is somewhat gray, complexion rather sallow, height and size medium. I have already alluded to him as an agreeable lecturer. He has a rich vein of humour, that breaks out from time to time to the infinite amusement of his audience.

Velpeau is one of the very first men of Paris, or of the world. His general scientific and professional attainments are of the highest order, and as a surgeon he is second to no man. As an author, his reputation is enviable. The recent edition of his great work on operative surgery, which professor Dunbar of Baltimore is now translating, being doubtless the best extant, on that subject. I have seen him perform almost all the capital operations—some of them frequently. He has recently exsected the lower jaw twice, and the upper jaw once—removed the uterus in two cases, and exsected several deep seated tumours of the sub-maxillary region. In diagnosis it would be difficult to find his superior. Some time ago, a man aged about twenty-five years, presented himself at the hospital with a large tumour of the scrotum; or rather a tumour *attached to the scrotum.* After due examination Velpeau humorously pronounced the fellow *pregnant with his little brother!*—in other words, that it was a case of superfœtation; and then proceeding to the operation, verified his diagnosis by exsecting the principal parts of a fœtus, to the great astonishment of the class. He remarked that the annals of surgery did not furnish such another case, in many of its features.

It is but proper to observe that a great proportion of his capital operations have been unsuccessful. This, however, is not the fault of the operator. Twenty years ago, Velpeau came to Paris without a profession, without money, and without friends—a poor boy from one of the provinces. A laudable ambition and untiring industry has made him what he is—a towering monument to the often repeated, but seldom appreciated adage, "labor vincit omnia."

The Hôpital de la Pitié is larger than La Charité. The number of its beds is near 600. It is finely situated in the face of the Garden of Plants, and includes several spacious courts, set in grass, and adorned by various species of shrubbery. In the year 1837, the last on which official documents have been published in relation to it, this establishment received into its medical wards 3,697 men, and 2,772 women. The number of surgical patients was 1,509. The mortality in the medical department was a little less than one in eleven—in the surgical about one to twenty-six and a half. The mean cost of each patient, per diem, is twenty-eight cents—the mean stay of the sick, or, in other words, duration of the diseases, was twenty days. The reason why the mortality of this hospital is less than that of the other similar establishments of the city, is probably to be found in its proximity to the Garden of Plants—a vast area planted in almost every species of the vegetable kingdom. St. George's of London is so situated as to be fanned by the air of St. James and Hyde Park, (the lungs of London) and is said, therefore, to be more salubrious than the other hospitals of that city. The most noted physicians of La Pitié are Piorry and Gendrin—they were rivals in the concours of last winter. Piorry was chosen, though I think it must be admitted that Gendrin is his superior. Lisfranc and Sanson are the surgeons. Lisfranc is a very large, noisy man, and takes great delight in abusing Velpeau, who perhaps is more amused at, than hurt by it. All the world knows that Lisfranc is a good surgeon, but not that he is more adroit and successful than any and every one besides. He attends to diseases of the uterus as a sort of speciality, and is far more successful in them (according to himself) than any other surgeon or physician. He uses the speculum uteri in every instance, by which he is enabled the better to diagnosticate the condition of the parts, especially of the os uteri. I have not room to say any thing of his treatment, nor do I know that there is any thing novel in it.

The Hospital St. Antoine was opened in the year 1796. It contains 300 beds. The number of admissions from 1804 to 1814 was 21,160, and the mean mortality one in five. In 1837 it was one in seven in the medical service, and one in seventeen in the surgical. The mean cost of patients, per diem, was one franc seventy-four centimes—about thirty-four cents—the number of admissions 3,495. The mean duration of their stay twenty-five days. Physicians, Kapeler and Guerard. Surgeon, Berard the elder.

The Neckar Hospital contains 400 beds. Its mortality from 1804 to 1814 was one in six. In 1837 it was one in six also, in the medical department, and one in seventeen in the surgical. The mean expense of each patient, per diem, two francs and thirty centimes. The number of admissions during that year 2,332, and the duration of their stay seventeen days. Physicians, Bricheteau and Trousseau. Surgeons, Civiale and Berard, the younger.

A few words in relation to Civiale and his operations for stone. He is a robust, fine looking gentleman, of perhaps fifty years of age. I have seen him operate very frequently. He used in every case the simple curved instrument of Heurteloup. Indeed I do not recollect that he ever introduced his own three branched instrument, more than once or twice. It would not be far from the truth to assert, that this very simple instrument is the best, and the only one necessary, in all the cases in which lithotrity is preferable to lithotomy. That there are a great many such cases no one now doubts, whose opinion upon the subject is entitled to consideration; and that there are many cases which require the knife, even Civiale admits; and sometimes resorts to it.

The Hôpital Cochin bears the name of its venerable founder. It contains only 100 beds. The number of its admissions during the year 1837 was 863. The mortality in the medical wards was one in seven, and in the surgical one in twenty-eight. Cost, per diem, for each patient two francs—thirty eight cents. Physicians, Blache and Briquet. Surgeon, Michon. M. Briquet is a warm advocate for mercurial ointment as a local application in smallpox; according to his experiment it prevents pustulation and its deprecated and indelible consequences.

The Hôpital Beaujon, situated in the northern suburbs of the city, contains 300 beds, and as considerable additions are being made to the buildings, this number will be greatly augmented. For the year 1837 its mortality was one in five. The mean expense for each patient one franc and forty-two centimes, and the mean duration of their stay twenty-three days. The great Louis is the principal physician. Marjolin and Robert are the surgeons.

Louis is a rather tall, handsome, staid-look ing personage, in spectacles, aged about fifty years.

The following are special Hospitals, or those devoted to particular classes of disease.

Hôpital Saint Louis. This is a very large establishment containing 800 beds, and devoted principally to the diseases of the skin; some chronic diseases, as rheumatism and scrofula, and cases of syphilis, are also received. These latter, however, according to Carmichael, should be classed with the exanthemata. There are given here each year about 50,000 baths, 40,000 fumigations, and 2,000 douches. The mean mortality from 1804 to 1814 was one in twenty-six, and the mean duration of the patient's stay thirty-two days. The number of admissions per annum has not varied for the last twenty-five years—being in 1812, 7,991, and in 1837, 7,940. The principal physicians are Gibert, Lugol, and Biett—the latter, however, has recently died. Richerand was recently attached to this hospital—he died during the last winter.

The Hôpital des Cliniques has been recently established. Its proximity to the school of medicine, renders it very convenient for clinical instruction, the principal object for which it was instituted; part of the wards are devoted to obstetrical practice. Rostan, who was at the head of the medical department, is removed to Hotel Dieu. I do not know who is in his place. J. Cloquet is the surgeon—Paul Dubois is the accoucheur, aided by some ladies almost as learned and skilful as himself. He performed the Cæsarian section last winter, and saved both mother and child.

Hôpital des Veneriens. Shortly after syphilis made its first appearance in Paris, it inspired such dread that a law was passed, that all strangers affected with it should quit the city, and be punished with death should they re-enter it uncured. Those who were confined to the hospitals were liable to the same punishment, if they ventured out without the authority of the physician. It was not surprising, that the feelings which gave existence to such a law, should also give rise to such an establishment appropriated to this class of maladies. This hospital contains 400 beds, all for males. The women have an establishment of their own, the Lourcine, and a prison, besides, for those of the frail sisterhood, who from time to time fall into the hands of the police. The mean mortality in this hospital for 1837 was one in two hundred and three; the mean stay of the patient thirty-two days; the number admitted 2,376. M. Ricord is the chief of this establishment, and no one (if every body is to be believed) is more worthy of the honour. A medal was awarded him by the Academy of Sciences, for his great work on venereal diseases. He has proven by numerous experiments the non-identity of gonorrhœa and syphilis; that matter taken from a syphilitic sore, produces a similar ulcer when inserted into the skin of any other part; while gonorrhœa matter will not. He regards the primary ulcer, or chancre, the 'fons et origo' of the secondary or constitutional form of the disease, and consequently cauterizes, or excises it as soon as possible. If this is not done without delay, says he, the constitutional contamination supervenes, and calls in most cases for the use of mercury. On this speciality, Ricord is perhaps the highest authority in the world. He is sometimes called to London in consultation on this and analogous diseases. He is an American by birth, and retains his partiality for his native land.—But I must pass on to the remaining hospitals, of which a very brief notice must suffice, as I have already extended this article to a greater length than I intended.

The Hôpital de Lourcine has already been alluded to as a female venereal establishment. The number of admissions in 1837 was 1,872; the mean mortality one in fifty, and the mean duration of their stay fifty days.

The Maison Royale de Santé is devoted to those who are able to defray a part of their expenses. The number of admissions for 1837 was 1,287; mean mortality one in six; mean duration of the disease twenty-three days. In this hospital the patient is of course more comfortably situated than in those in which he contributes nothing to his maintenance and medical attention.

Hôpital des Enfants Maladies. This is a large and airy establishment, situated in the southwestern suburbs of the city. The two sexes are here received, aged from one year to fifteen. It contains 560 beds. The mean mortality of this hospital has always been very great—nearly one in four. The number of its inmates is at present more than 3,0C0.

It is here that M. Guérin, the great orthopedist, performs so many operations for club-foot. His method is to cut all the tendons concerned in the production of the deformity.

The following establishments are termed Hospices. They are designed as asylums for infancy, age, and those affected with incurable diseases:

The Hospice d'Accouchement is devoted to pregnant women who have attained their eighth month. The number of beds is 433. It is well ventilated, and has several fine promenades; its wards are neat and well regulated. In short, the French seem to think that women waiting for the pains of parturition at the Hospice d'Accouchement are in no unenviable condition.

Hospice des Enfans Trouvés, or Foundling Hospital. Until within a few years the mortality of this asylum was almost incredible, being some years four out of five, and even now, after all that vigilance and improved regulations can do, has been done, it amounts to almost one-third. The number at present is not far from 5,000.

Hospice de Salpétrière. This is one of the most splendid and extensive asylums in the world. It is designed for indigent women who have attained the age of seventy years, and the poor of the same sex who are afflicted with mental derangement, idiocy, epilepsy, &c., &c., of all ages. The number of beds for the old and indigent is 5,000, conveniently distributed in vast ranges of buildings appropriated to this class of inmates. The number of the deranged, idiotic, epileptic, &c. varies from one thousand to twelve hundred. They are so disposed of, as not to incommode each other, or disturb the tranquillity of their neighbours—the old women. The proportion of cures is about one-third. Harsh means, either of a moral or medical character, are never resorted to. The remedies principally used, besides kind treatment, are baths, douches, mild purgatives, exutories, &c., with or without isolation; all these means are of course varied according to circumstances.

The Hospice de Bicêtre, situated in the country a little more than a mile from the city wall, differs in destination from Salpétrière only as to sex. Here males only are admitted, of whom also two grand divisions are made—the aged and the alienated. Of the former there are about 3,000, of the latter 8,000. Bicêtre is situated on elevated ground surrounded by cultivated fields, and commands a fine view of the southern portion of the city.

I must bring this notice to a close with a mere mention of the seven remaining Hospices. They are consecrated to the incurable, the indigent, and the aged, and are designated as follows: Hospice of incurable men, Hospice of incurable women, Hospice des ménages, H. de La Rouchefoucauld, the Institution of St. Perine, H. St. Michel, and the H. de Villas, making in all seven general hospitals, five special hospitals, and ten hospices.

